# Genotype‐by‐environment interactions drive the maintenance of genetic variation in a *Salmo trutta* L. hybrid zone

**DOI:** 10.1111/eva.13307

**Published:** 2021-10-30

**Authors:** Dorinda Marie Folio, Jordi Gil, Arnaud Caudron, Jacques Labonne

**Affiliations:** ^1^ Université de Pau et des Pays de l’Adour UMR INRAE‐UPPA Ecobiop Saint‐Pée‐sur‐Nivelle France; ^2^ SCIMABIO Interface Thonon‐les‐Bains France; ^3^ UMR CARRTEL INRAE USMB Thonon‐les‐Bains France; ^4^ Conservatoire des Espaces Naturels Rhône‐Alpes Vogüe France

**Keywords:** embryonic survival, hybridization, introgression, maternal effect, postzygotic selection, reproductive isolation

## Abstract

Allopatric gene pools can evolve in different directions through adaptive and nonadaptive processes and are therefore a source of intraspecific diversity. The connection of these previously isolated gene pools through human intervention can lead to intraspecific diversity loss, through extirpation of native populations or hybridization. However, the mechanisms leading to these situations are not always explicitly documented and are thus rarely used to manage intraspecific diversity. In particular, genotype‐by‐environment (GxE) interactions can drive postzygotic reproductive isolation mechanisms that may result in a mosaic of diversity patterns, depending on the local environment. We test this hypothesis using a salmonid species (*Salmo trutta*) in the Mediterranean (MED) area, where intensive stocking from non‐native Atlantic (ATL) origins has led to various outcomes of hybridization with the native MED lineage, going from MED resilience to total extirpation *via* full hybridization. We investigate patterns of offspring survival at egg stage in natural environments, based on parental genotypes in interaction with river temperature, to detect potential GxE interactions. Our results show a strong influence of maternal GxE interaction on embryonic survival, mediated by maternal effect through egg size, and a weak influence of paternal GxE interaction. In particular, when egg size is large and temperature is cold, the survival rate of offspring originating from MED females is three times higher than that of ATL females’ offspring. Because river temperatures show contrast at small scale, this cold adaptation for MED females’ offspring constitutes a potent postzygotic mechanism to explain small‐scale spatial heterogeneity in diversity observed in MED areas where ATL fish have been stocked. It also indicates that management efforts could be specifically targeted at the environments that actively favor native intraspecific diversity through eco‐evolutionary processes such as postzygotic selection.

## 1 INTRODUCTION

Admixture of previously isolated gene pools, through human intervention, often leads to gene flow and hybridization. Indeed, many ecological interactions are observed between individuals of distinct species or populations, such as between wild and domesticated individuals (Ryman et al., 1995; Utter, 2004) that escaped or were willingly introduced in natural populations (Drinan et al., 2015; McGinnity et al., 2003; Reed et al., 2015). Based on these individual interactions, hybridization can have various impacts (Epifanio & Nielsen, 2001; Genovart, 2009; Todesco et al., 2016). In some cases, hybridization may increase genetic diversity, possibly via evolutionary rescue (Stelkens et al., 2014; Todesco et al., 2016). However, in most cases, hybridization has negative impact on native population demography and diversity (Rhymer & Simberloff, 1996; Todesco et al., 2016). Indeed, demographic, pathogenic, and genetic consequences reducing the fitness of native populations are often observed following admixture (Budy et al., 2013; McGinnity et al., 2003; Závorka et al., 2018 but see Stephens et al., 2020). In the worst case, extinction by hybridization and introgression can occur (Rhymer & Simberloff, 1996; Seehausen, 2006). The degree and outcomes of hybridization in natural environments will depend upon existing reproductive barriers, which may limit gene flow yet not always fully prevent it (Bettles et al., 2005; Mallet, 2005).

Notably, following the admixture of two gene pools, postzygotic barriers can lead to different outcomes regarding hybrid fitness and hence population diversity. On the one hand, hybrid fitness can be reduced compared to “native” genotypes due to outbreeding depression. This mechanism is sometimes expected to act as a purifying force that could favor the conservation of native—possibly adaptive—genetic variation (Broadhurst et al., 2008; Hansen et al., 2009; Kronenberger et al., 2018; Ruzzante et al., 2004). Commonly applied management practices, such as the implementation of genetic refuge, native individual translocation or restocking (Caudron et al., 2006, 2011, Caudron, Champigneulle, et al., 2012; Grobler et al., 2011), or any practices involving admixture, often rely on this assumption. However, outbreeding depression may also be associated with the genetic swamping of adapted genes (Allendorf et al., 2001; Rutherford et al., 2019), which could counter‐effect these management practices. An alternative conservation measure might be to remove “non‐native individuals” (Bohling, 2016; Caudron & Champigneulle, 2011; Guay et al., 2014; Muñoz‐Fuentes et al., 2007), but this can only be viable when hybridization is restricted in space or not frequent (Genovart, 2009). On the other hand, gene flow can help to build new genetic linkages that are equally or more adaptive than “native” ones (“admixture effect”, Zalapa et al., 2009). A possible consequence is the general increase of fitness in the receiving populations (i.e., “genetic rescue,” Fitzpatrick et al., 2019), at the detriment of pure “native” genetic variation. Management options to conserve such native variation, in that case, are limited (Genovart, 2009). When pure native populations still exist, one solution might be to isolate them to prevent any further gene flow (Bohling, 2016; Guay et al., 2014).

Mounting evidence indicates that these management practices generally fail at conserving native diversity after admixture had already occurred (Gil et al., 2016; Muñoz‐Fuentes et al., 2007; Vincenzi et al., 2012). This is possibly due to the lack of knowledge regarding reproductive isolation mechanisms (Bajec et al., 2015; Berrebi, Poteaux, et al., 2000; Iacolina et al., 2019; Taillebois et al., 2020), which could help to build more efficient and evolutionary‐inspired management strategies. Identification of both pre‐ and postzygotic reproductive barriers should thus be a prerequisite to any management decisions to avoid counter‐productive results. Indeed, since admixed populations had previously evolved separately in different environments, they may have diverged genetically and phenotypically, possibly due to local adaptation. Fitness variation in relation to some environmental factors can thus be expected: Some genotypes may achieve better fitness in some environments than other genotypes. Information on the environmental factors that may contribute to reproductive isolation will help in understanding and predicting the outcomes of hybridization in specific environments. This could then be used to implement new management practices (Epifanio & Nielsen, 2001; Genovart, 2009; Todesco et al., 2016) to either avoid admixture (if applied soon enough), lesser the extent of introgression, and favor “native” genes conservation when admixture has already occurred (Caudron, Champigneulle, et al., 2012), or to favor diversity when it enhances the adaptive potential of the population (Chan et al., 2019).

It is therefore paramount to investigate and assess the strength and direction of such genotype‐by‐environment (GxE) interactions to (i) maximize beneficial genetic and phenotypic variation as it represents a resilience mechanism against environmental change (Cook & Sgrò, 2018; Jump et al., 2009; López‐Pujol et al., 2012; Violle et al., 2012) and to (ii) develop eco‐evolutionary‐based approaches allowing to target specific operations of conservation where they will be the most effective (Caudron, Vigier, et al., 2012; Chan et al., 2019; Genovart, 2009; Todesco et al., 2016).

Salmonids embody the above‐mentioned situation since they evolved mostly in allopatry during the Pleistocene glaciations, where geographical reproductive isolation allowed potential divergent evolution to operate (Bernatchez, 2001). Among them, *Salmo trutta* L. (Brown trout) holds at least five genetically and phenotypically distinct lineages (Bernatchez, 2001) that evolved in allopatry for 0.5 to 2 million years during the glacial era, where separated populations colonized different areas across Europe (García‐Marín et al., 1999), although hybridization may have occurred at some points (Hashemzadeh Segherloo et al., 2021). However, since the discovery of artificial reproduction at the end of the 19th century, *Salmo trutta* became the most introduced fish species in the world (Lowe et al., 2000). As one of the consequences, the distinct lineages have been forced back in sympatry. The Atlantic (ATL) lineage, in particular, has been intensively used for fish farming and intensive river stocking to sustain recreational fishing (Beaudou et al., 1994; Berrebi, Povz, et al., 2000; Caudron & Champigneulle, 2007; Krieg & Guyomard, 1985; Largiadèr et al., 1996; Launey et al., 2002; Presa et al., 1994). In the Mediterranean area, where the Mediterranean (MED) lineage has evolved, the resulting situation displays a mosaic of outcomes, from total extirpation of the MED lineage to various degrees of hybridization (HYB), with some rare patches of pure MED gene pools remaining (Caudron, Champigneulle, et al., 2012). The reason for this spatial heterogeneity and diverse levels of intraspecific diversity cannot be solely explained by management practices (i.e., intensity of restocking using ATL lineages, Gil et al., 2016). However, up until now, no postzygotic isolation mechanism has been studied in natural populations presenting contrasted environments.

In the present study, we investigated patterns of offspring survival at egg stage in *Salmo trutta*, based on parental genotypes—using markers related to original MED and ATL lineages—in interaction with river temperature to detect potential GxE interactions. Selection can indeed act very strongly during early stages of development in salmonid species, notably as a function of temperature (Huuskonen et al., 2003; Ojanguren & Braña, 2003; Régnier et al., 2013), a factor that can show contrast at small scales (Brown & Hannah, 2008; Daigle et al., 2016). Offspring from ATL lineage are expected to be adapted to relatively warm temperatures (8–10°C) for prehatching survival as already demonstrated (Jungwirth & Winkler, 1984; Ojanguren & Braña, 2003; Régnier et al., 2013), a thermal range often encountered in their distribution area. For the MED lineage, which evolved experiencing a wider range of contrasting temperatures (Daigle et al., 2016), we hypothesize that their offspring should at least outperform ATL offspring in cold water rate (4–6°C), since ATL offspring display very low survival at such temperatures (Ojanguren & Braña, 2003; Régnier et al., 2013). To assess the real importance of temperature on postzygotic selection, our experiment was performed directly in natural environments where substantial temperature contrasts are observed during winter (Burt et al., 2011). In that way, other uncontrolled known and unknown factors can interactively affect survival, and any significant effect of temperature variation on offspring survival can therefore be deemed as an important driver of natural evolution (Anderson et al., 2014; Genovart, 2009). Based on our results and interpretation of GxE interactions, we propose new evolutionary‐based guidelines for management strategies, to either enhance native diversity conservation or maintain adaptive potential in spatially variable environments.

## 2 MATERIALS AND METHODS

### 2.1 Genitors sampling and study sites

**FIGURE 1:**
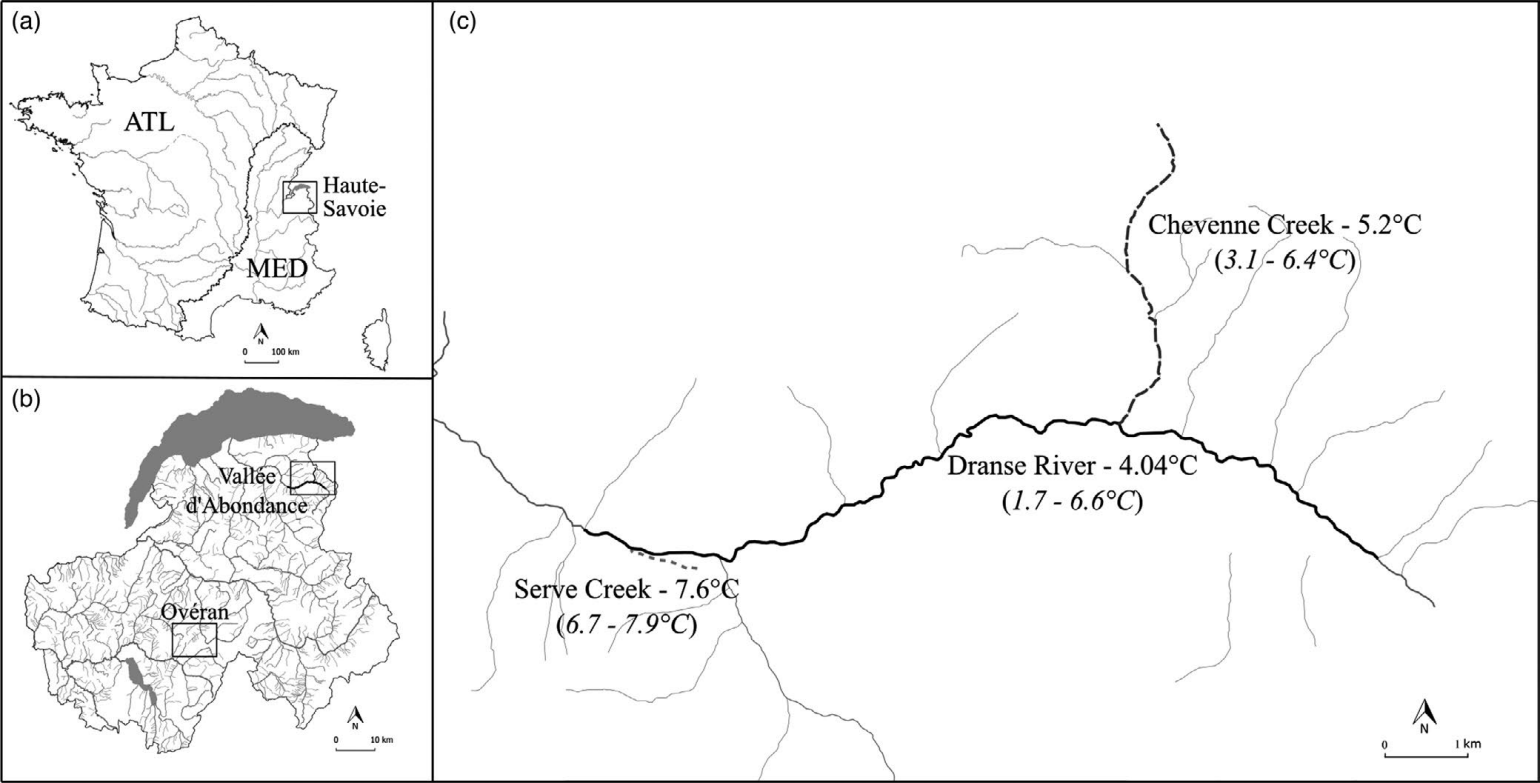
Map of France with the delimitation between the Atlantic (ATL) and the Mediterranean (MED) basin as well as a focus on the Haute‐Savoie region (a) where river locations for adult *Salmo trutta* sampling and egg incubation are indicated (b) and topography of the Dranse d’Abondance system with our three thermally contrasted rivers (c). Mean water temperatures are indicated and in brackets are minimum and maximum recorded water temperatures over the study period

The present experiment was performed in the Haute‐Savoie region, France, part of the Rhone river basin, originally occupied by the MED lineage of *Salmo trutta*. In this region, a century of ATL *Salmo trutta* introduction has generally led to the presence of ATL individuals located upstream, HYB individuals located in the intermediate parts of the rivers, and MED individuals located downstream in the confluence areas (Caudron et al., 2009). Adult *Salmo trutta* were sampled from the Overan Creek, a four‐kilometer long tributary of the Borne River (46.55′57.68N″;6°24′07.40E″, Figure 1a,b) that harbors sufficient genetic variation required for the present study. We focused our sampling on this single river to avoid confounding effects of local adaptation (see Drinan et al., 2012), although local adaptation at such scale is not usually found (Stelkens et al., 2012). To account for differences in maturation dates among individuals, adult mature fish were captured on five different occasions, spread between December 8, 2013, and January 15, 2014—throughout reproduction period—using electric fishing. Availability of gametes was assessed by palpation, so to ensure successful fertilization and realization of the protocol. It was paramount to limit the captivity duration of genitors to a few days so to prevent any mortality and to safely proceed to successful fertilization of the desired progenies. As a consequence, progenies were obtained before knowing the exact genotypes of genitors. Adults were thus selected on robe criteria, which can be used to some extent as proxies of genetic lineages characterization (Data S1).

*In fine*, we selected 23 fecund males and 9 fecund females, hoping to obtain a substantial genetic contrast in origins for our fertilization protocol (individual characteristics for parents are provided online: https://doi.org/10.15454/KB0NL3). The selected individuals were placed in oxygenated water and transported to the experimental fish farming installation in Thonon‐les‐Bains (INRAE, E74 300‐4). A few days before fertilization, fish were kept unfed in oxygenated tanks and received two treatments against saprolegniasis.

Three neighboring rivers were then selected in Haute‐Savoie, to later deposit eggs during their incubation period, in order to represent a thermal contrast to test for GxE interactions (Figure 1b,c): the Dranse River (46°16′52.94N″;6°42′39.55E″, slope = 4%, altitude at source = 1500 m) and two of its tributary streams, the Chevenne Creek (46.17′54.04N″;6°47′22.09E″, slope = 10%, altitude at source = 1250 m), and the Serve Creek (46.16′49.60N″;6°42′40.83E″, altitude at source = 840 m). They had respectively low (4.04°C), intermediate (5.2°C), and high (7.6°C) mean water temperatures (averaged over December 1, 2013 to March 30, 2014).

### 2.2 Fertilization protocol

Three fecund females were selected per expected genotypes (ATL, HYB, MED) based on robe criteria, and their eggs were collected for experimental fertilization. Females, however, produced eggs of variable sizes (Data S2). Therefore, in each clutch, 30 eggs were photographed and measured to estimate mean egg size in order to control for maternal investment via egg‐size effects. Each female's clutch was then divided into four batches: Three batches were destined to be placed in the three rivers previously selected for their contrasted thermal regimes, and the last batch was left in the fish farming installation (Thonon‐les‐Bains, INRAE, E74 300‐4) to simply control for fertilization success. Each batch was again divided into three sub‐batches in order to be fertilized by semen from either expected MED, expected HYB, or expected ATL males (also selected on robe criteria). A total of 81 egg sub‐batches were available for our field experiment, 27 per thermal environment, representing the nine possible crosses between MED, HYB, and ATL males and females expected genotypes (Data S3). Because one male's semen was not always sufficient to fertilize all the sub‐batches for all the females in all conditions, several males with the same robe criteria could be used for different sub‐batches. However, males’ semen was never mixed together, so to keep track of the parental design: each sub‐batch represented a full‐sib family, allowing us to disentangle parental effects from the desired genotypic effects.

### 2.3 Experimental design

Following fertilization, each sub‐batch (between 20 and 44 fertilized eggs) was placed in an incubation box (12 cm long, 5 cm diameter), which protects eggs from predation, filled with gravel (5–20 mm diameter) matching river substratum. Incubation boxes were then transported to each river site. Eggs were slowly brought to river temperature and incubation boxes were then buried in the river substratum, to mimic nest‐building behavior, at depth matching the natural range of variation observed in *Salmo trutta* (Gauthey et al. (2015); depth ranging between 5 and 15 cm, average = 10 cm depending on substratum availability), still within fertilization day. Pine sticks were placed in the substratum near incubation boxes to monitor for possible under‐gravel anoxia events (Marmonier et al., 2004) that could have affected egg survival during incubation (Bloomer et al., 2019; Roussel, 2007; Winnicki, 1967).

A total of 81 incubation boxes (Data S3) were thus placed in situ between December 9, 2013, and January 16, 2014, and removed at approximately 400 degree‐days—that is daily accumulated temperature required for eggs to hatch (Killeen et al., 1999). On‐site temperature loggers were used to record hourly temperatures and forecast the embryogenic development until hatching stage (about 400 degree‐days). Incubation boxes were dug out of the rivers from beginning of February to end of March 2014 as burial happened at different dates (due to genitors’ availability for fertilization) and development time varies according to thermal regimes. For each recovered box, the number of surviving vesicled‐fry was counted and occurrence of saprolegnia noted. Egg sub‐batches, left at the fish farming installation, indicated that fertilization rates were high (ranging between 85% and 100%).

### 2.4 Genotypic studies

To assess parental genotype, genitors pelvic fins were clipped and analyzed at six diagnostic markers (four single nucleotide polymorphism loci: OMM1144, OMM1105, OMM1154 and OMM117, unpublished data and two microsatellites: str541 and str591, Gharbi et al., 2006) perfectly differentiating ATL and MED lineages in *Salmo trutta* (Guyomard & Caudron, 2008, unpublished data). Offspring genotype was not assessed. A genotypic score was attributed to each parent and determined based on the allele number of each lineage on all loci. An individual presenting 12 MED alleles was given a genotypic score of 1, deemed as a pure MED genotype, whereas an individual with 12 ATL alleles was given a genotypic score of 0, hence deemed as a pure ATL genotype. All other intermediate scores were referred to as HYB genotypes. Although genotypic scores were obtained *posterior* to fertilization, they were indeed strongly correlated to robe criteria, ensuring that our fertilization protocol allowed us to cross parents of the desired genotypic scores (Data S4).

### 2.5 Statistical analyses

We analyzed offspring survival probability *p* (ratio of the surviving fry number conditional on the initial egg number placed in each incubation box) as a function of parental GxE interactions using mixed‐effects logistic regression. To do so, we tested for linear effects of maternal (*MG*) and paternal (*PG*) genotypes—that is genotypic score—in interaction with a linear effect of average temperature during the incubation period (*T* ^°^) for the incubation box *l*. Since rivers presented contrasted temperatures, the required time to reach 400 degree‐days varied significantly among incubation boxes. Therefore, we decided to control for the number of days spent in the incubation box before hatching (Days). We also considered egg size (*E*), as well as the occurrence of saprolegnia (*S*), to respectively control for maternal investment and disease‐related effects on offspring survival. We tested for other targeted interactions of interest between both parental genotype and temperature, and between egg size, maternal genotype, and temperature. Pseudo‐replication related to the identity of males (*α_i_*) and females (*β_j_*) was treated using random effects. We also included a random effect for the three rivers (*γ_k_*) to effectively disentangle pure temperature effects from other unknown environmental factors.

$$\begin{array}{cc} \text{logit}\left(p_{i,j,k,l}\right) & =\delta *MG_{j}+\varepsilon *PG_{i}+\zeta *T_{l}^{\circ }+\eta *{\text{Days}}_{l}+\theta *E_{j}+\kappa *S_{l}\;\;\;\;\left(\text{Basic factor effects}\right)\\ & \;+\lambda *MG_{j}*PG_{i}+\mu *T_{l}^{\circ }*MG_{j}+\nu *T_{l}^{\circ }*PG_{i}+\xi *E_{j}*T_{l}^{\circ }+\rho *E_{j}*MG_{j}\;\;\;\left(\text{Interactions}\right)\\ & \;+\sigma *E_{j}*T_{l}^{\circ }*MG_{j}+\varphi *T_{l}^{\circ }*MG_{j}*PG_{i}\;\;\;\left(\text{Three - way interactions}\right)\\ & \;+\alpha_{i}+\beta_{j}+\gamma_{k}\;\;\;\left(\text{Random effects}\right) \end{array}$$

with δ,ε,ζ,η,θ,κ,λ,μ,ν,ξ,ρ,σ,φ the model parameters to be estimated.

Statistical inference was conducted in the Bayesian framework using Markov chain Monte Carlo (MCMC) techniques as implemented in JAGS software (Plummer, 2003). We used noninformative normal prior distributions for the fixed effects (0.0, 0.0001) and noninformative gamma prior distributions (0.001, 0.001) for precision parameters of the random effects. The model code and data are available online (https://doi.org/10.15454/KB0NL3). To approximate the joint posterior distributions of all unknown quantities of the model—that is parameters—one MCMC with 10,000 iterations was used after a 5000 iterations burn‐in period and after checking its convergence on three chains by applying the Gelman‐Rubin test (Gelman & Rubin, 1992). We also calculated the percentages of the posterior distribution above and below zero for each parameter and considered a parameter to be significantly different from zero when one of these percentages was below 5%.

## 3 RESULTS

Out of 81 incubation boxes placed in natural rivers, 16 could not be recovered because they were scoured at high flow (a natural phenomenon). Among the 65 recovered boxes, some parent contributions could not be represented in some rivers, either due to nest scouring or to the lack of gametes and possibly reducing overall available genetic variation for selection. Despite this, nearly all possibilities of parental genotypic score combinations were present in all three rivers (except for crosses involving HYB females, Data S5). Pine sticks visual analysis revealed no anoxia event, but footprints of saprolegnia contamination were observed in 32 of the recovered boxes. The parameter estimates indicated that the occurrence of saprolegnia had a significant negative effect on survival probability (Table 1). The number of days before hatching also had a significant effect, wherein longer durations were correlated with better survival.

**TABLE 1:** Parameter estimates of the mixed‐effects logistic regression model. Posteriors mean, 95% credible interval, and percentage above or below zero (Percentage/0) are provided for the controlled and tested effects, as well as interactions

Effects	Parameter	Median	95% credible interval	Percentage/0
Controlled for	Days	0.082	[0.033; 0.132]	0.24^**^
	Saprolegnia (*S*)	−0.570	[−0.860; −0.278]	0^***^
Tested	Egg size (*E*)	−3.35	[−4.669; −1.768]	0^***^
	Temperature (*T*°)	−0.749	[−1.38; −0.066]	1.6^*^
	Maternal genotype (*MG*)	−15.85	[−26.08; −5.02]	0.14^**^
	Paternal genotype (*PG*)	1.179	[−0.565; 2.977]	9.9
Interactions	$T^{\circ }*MG$	2.574	[0.759; 4.215]	0.28^**^
	$T^{\circ }*PG$	−0.271	[−0.563; 0.016]	3.279^*^
	MG∗PG	−0.393	[−3.367; 2.295]	39.75
	E∗MG	4.325	[1.772; 6.785]	0.02^***^
	$E*T^{\circ }$	0.494	[0.256; 0.705]	0^***^
Three‐way interactions	$E*T^{\circ }*MG$	−0.676	[−1.054; −0.251]	0.02^***^
	$T^{\circ }*MG*PG$	0.0543	[−0.399; 0.550]	59.31

Note

Values significance for the last entry were positively represented by * symbols.

**FIGURE 2:**
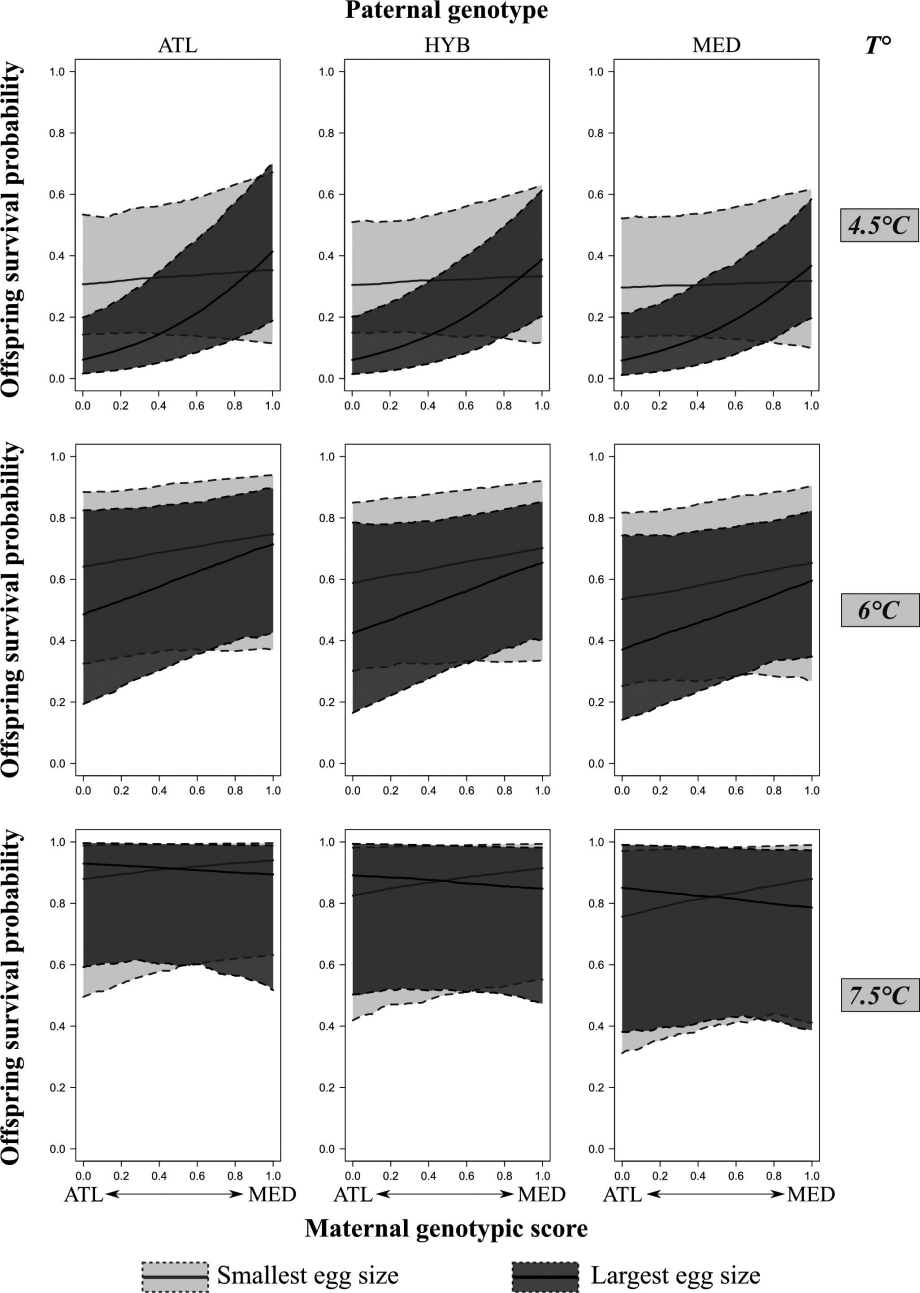
Predictions of offspring survival probability at low (4.5°C), medium (6°C), and high (7.5°C) temperatures as a function of maternal genotypic score, for ATL (0), HYB (0.5), and MED (1) paternal genotypes. Values for the smallest (3.5 cm) and largest (5.25 cm) eggs found in our sample are represented separately to account for egg size variation. Solid lines are posterior means, while dotted lines are 95% credible intervals. Predictions were performed using male ID 23, female ID 9, and the Dranse River as random effects. Saprolegnia effect was discarded from the predictions

Most of the factors of interest and most of their interactions (Table 1) were significantly different from zero, pointing at complex patterns of variation in offspring survival probability. It is noteworthy that the only nonsignificant interactions were those including both maternal and paternal genotypes. All other interactions including maternal genotype and temperature were significant, indicating that GxE interactions occurred. Figure 2 describes the predicted distribution of survival probability of offspring, as a function of parental genotypes, temperature, and egg size, allowing us to envision the shape and strength of the GxE interactions. In general, offspring survival was higher at warm temperatures than at cold temperatures. For the largest eggs (5.25 mm), at cold (4.5°C) temperature, the survival probability for offspring of MED females was at least three times higher than that of offspring of ATL females, but this difference nearly disappeared for the smallest eggs (3.75 mm). At intermediate temperature (6°C), offspring from MED females still had a slightly higher survival probability than offspring from ATL females, whatever the egg size. At warm temperature (7.5°C), the relationship between maternal genotype and survival disappeared, as well as the effect of egg size. The paternal genotype effect, though weak, was mainly expressed at high and intermediate temperatures, favoring offspring sired by ATL males, hinting at another potential GxE interaction. The addition of the female and male GxE interactions implied that offspring survival was often the highest for hybrid descendants sired by ATL males and MED females, whereas other hybrids (different crosses) displayed intermediate survival in most cases.

The distribution of random effects indicated no strong additional female effects on offspring survival, whereas one male (ID = 12) had a particularly negative effect on offspring survival. Additionally, random effects for rivers showed that offspring placed in the Serve Creek had lower survival than those placed in the Chevenne Creek and the Dranse River, unconditional on water temperature (Data S6).

## 4 DISCUSSION

This study expands our knowledge of GxE interactions and reproductive isolation mechanisms between genetically distinct lineages brought in sympatry, by showing that fitness variation in hybrid zones following admixture can be strongly related to genetic origins and can be extremely spatially variable. Of particular interest is the fact that our experiment was performed in natural environments using wild genitors, an approach that allows us to measure the effect of the factor of interest (here, water temperature) without removing all other natural sources of variation.

### 4.1 Evidence of gene‐by‐environment interactions

As hypothesized, temperature contrasts between rivers substantially affected offspring survival in our experiment. Offspring generally had higher survival at the highest temperature studied, which falls within the range of optimal temperatures for the survival of *Salmo trutta* during prehatching stage (Jungwirth & Winkler, 1984; Ojanguren & Braña, 2003). Ojanguren and Braña, (2003) reported an average 70% decrease in ATL offspring survival (at egg stage) for a decrease of temperature from 7.5 to 4.5°C, very much in line with our findings for ATL females. In particular, for the ATL lineage, offspring from larger eggs performed worse than those from smaller eggs at low temperatures, confirming previous experimental results in captivity (Régnier et al., 2013). However, for the same egg size, offspring from MED females had a three times higher survival probability than offspring from ATL females at cold temperatures. This constitutes a major selective advantage in cold environments for carriers of MED maternal genes. It is also noteworthy that, generally, egg size is positively correlated to female body size in salmonids, as is fecundity (Lobon‐Cervia et al., 1997): Large females lay large and numerous eggs and therefore can massively contribute to population growth. To our knowledge, there is no documented difference between lineages regarding this size‐dependent allocation strategy. Thus, the present GxE interaction, mediated by maternal investment, we here uncovered may fundamentally alter the genetic structure of populations through boosted population growth by strongly favoring MED maternal ascendency in cold environments.

Focusing on the effects of paternal genotype now, we also found a GxE interaction involving temperature contrast. Genes from ATL males tended to slightly improve offspring survival, but more so at warm temperatures. The strength of this effect however was much lesser than the above‐mentioned female GxE interaction, a somewhat logical outcome since maternal effects are often stronger than paternal effects during earlier phases of development (Burton et al., 2006; Burton et al., 2020; Huuskonen et al., 2003; Régnier, Labonne, et al., 2012). The fact that we did not find any significant interactive effect between paternal and maternal genotypes seems to preclude any assumption regarding possible benefits of increased heterozygosity (Dahl et al., 2006; Edmands, 2007; Fraser et al., 2008; Meldgaard et al., 2007; Weatherhead, 1999; Wells et al., 2019), such as higher diversity at functional loci like MHC (Jacob et al., 2010; Landry et al., 2001; Turner et al., 2009 but see Labonne et al., 2016; Tentelier et al., 2017). Therefore, ATL males may carry good genes—for some environments—that MED males do not have. One possible speculation to explain this is to turn to the SEX locus in salmonids (Yano et al., 2012, 2013), which is only present in males: Some beneficial genes could be segregated with this locus only in the ATL lineage.

### 4.2 Potential consequences on diversity and reproductive isolation

It is noteworthy that even if our experiment was realized on a small spatial scale, we may have found footprints of adaptation to temperature wherein some genotypes performed better in cold environments than others; where other studies (also focusing on the embryonic stage) involving a single lineage failed to do so (Clark et al., 2013; Stelkens et al., 2012). While evidence for adaptation to cold conditions in high altitudes was demonstrated at the species level, for *Salmo trutta* (Jungwirth & Winkler, 1984) or *Salvelinus alpinus* (Arctic char, Huuskonen et al., 2003), lineage‐related adaptation to temperature is to our knowledge not documented. Low temperatures are often reached in alpine systems, such as the Dranse d’Abondance, where the MED lineage evolved since the postglacial period (Bernatchez, 2001). This potential lineage‐related adaptation to cold conditions indicates in our case that despite several generations in sympatry, gene flow has not erased the link between the set of 6 diagnostic markers that were previously designed on the two separate lineages and genes under potential selection (de Lafontaine et al., 2015; Fitzpatrick et al., 2020; Lamaze et al., 2012). Of particular interest is the fact that robe criteria were well correlated to our genotypic score, whereas recent results indicate that such approach may still miss a large part of the recent genetic admixture (Saint‐Pé et al., 2019). This indicates that additional investigations of lineage differences regarding adaptation to cold (MED) or warm (ATL) temperatures could produce even more insightful data to further our understanding of intraspecific diversity dynamics in hybrid zones and to understand how thermal environment could control for postzygotic reproductive isolation (Leitwein et al., 2016). Given the recent progress in the taxonomic status of Brown trout (Hashemzadeh Segherloo et al., 2021), it is also likely that such hybridization events, through their consequences on fitness variation, are central to the evolution of the whole species (or species complex).

Temperature variation range itself is very heterogeneous at different spatial and temporal scales in mountain hydrosystems (Brown & Hannah, 2008; Daigle et al., 2016). It was also the case on our field study sites, wherein the three rivers were connected, within a few kilometers, but presented contrasted thermal regimes. These temperature contrasts cannot be summarized by the altitude gradient: Geothermal influence, as well as distance to source or exposition to wind and light, can also strongly condition water temperature dynamics. This, in conjunction with temperature‐based GxE interactions, could explain some of the hybridization patterns that are observed in the Haute‐Savoie region and elsewhere. For instance, some pure MED populations appear to have much greater fitness than some pure ATL populations in some river stretches, whereas in other stretches, they are extremely vulnerable to introgression and have been extirpated (Gil et al., 2016). Likewise, Largiadèr and Scholl (1996) failed to find frequent hybrids in the Doubs population and concluded to potent reproductive barriers at work there: This could be related to cold temperature adaptation.

Our findings may also explain the predominance of native maternal ascendance in hybrid zones. In the Chevenne creek for instance—one of the three rivers tested here—mitochondrial lineages exclusively point at the MED lineage (Gil et al., 2016). Such predominance of the native maternal ascendance is also found in other areas (Poteaux et al., 1998) and in other hybrid zones in *Salmo trutta* for a different lineage (Pujolar, Lucarda, et al., 2011; Pujolar, Vincenzi, et al., 2011) or in other species (Bonnet et al., 2017; Schwartz et al., 2004; Taillebois et al., 2020). When combined with our results, this indicates that maternal effects (genetic and nongenetic) may have a preponderant role in the dynamics of hybridization, notably because they are especially present during the first stages of life (Burton et al., 2006; Burton et al., 2020; Giesing et al., 2011; Régnier, Bolliet, et al., 2012; Régnier, Labonne, et al., 2012; Shu et al., 2016). Cold adaptation, in particular, is partly mediated through physiological mechanisms, which often involve mitochondrial functions that are hence linked to maternal ascendancy (White et al., 2012) and can differ between lineages (Kavanagh et al., 2010). Additionally, in the present experiment, females did not choose their spawning habitat, whereas it can also further improve offspring fitness (Armstrong et al., 2003; DeVries, 1997; Gauthey et al., 2015; Riedl & Peter, 2013), possibly reinforcing GXE interactions effects.

Finally, it is also important to consider to what extent our experimental protocol can be compared to mating patterns occurring in wild populations with regard to genotypic variation (Maan & Seehausen, 2011). Gil et al. (2015) found that female sexual preference in MED/ATL hybrid zones is generally aimed at dissimilar males with respect to lineage‐related robe criteria. This implies that homogamous mating—with respect to lineage—may not be very frequent. However, the fact that we found pure ATL or MED genotypes among our genitors from the Overan River also evidences the possibility of some homogamous mating. It is therefore likely that most of the combinations envisioned in our protocol can also occur in wild populations, although some of them might be more frequent.

### 4.3 Implications for intraspecific diversity management

The effects of human‐induced environmental perturbation on evolutionary processes often result in the erosion of reproductive isolation between previously separated lineages (Grabenstein & Taylor, 2018). Elucidating the evolutionary mechanisms controlling this erosion, however, can help to formulate sound management decisions. Based on the present results, we advise allocating management effort where they can bring the higher overall benefit. For managers interested in protecting “native” MED genetic variation, some environments should be particularly targeted for conservation actions: For instance, cold environments may naturally and very efficiently select for native genotypes in our example—or at least may help to preserve the native MED female lineage. This opens a path for managers to target sanctuaries based on thermal monitoring. It suggests that baseline information on climatic parameters and spatial projection related to the local effects of climate change should be performed by managers. Using such spatial mapping of the factor of interest (here, water temperature) at small scale and considering the spatial distribution of available genetic variation (Razgour et al., 2019) may considerably help to forecast the evolution of intraspecific dynamics. Management strategies aimed at conserving native diversity, such as selective removal of non‐native phenotypes or genotypes, introduction of native genotypes, could therefore be targeted at the right environments and become much more efficient. In short, knowledge of GxE interactions opens a path for a cost‐effective approach to native diversity conservation.

On the contrary, in environments where hybrids already occur, with intermediate‐to‐high temperatures, it might be counter‐productive to attempt to eradicate hybrids, since they might have an equal or better fitness than native MED individuals and also because reducing their frequency might, in fact, increase their mating success through heterogamous sexual preference (Gil et al., 2015) reinforced by negative frequency dependence (i.e., preference for rare phenotype, Hughes et al., 1999; Kokko et al., 2007). The interplay between heterogamous mating preferences with regard to lineages and postzygotic GxE interactions is however likely to generate ample genetic variation in hybrid zones. Such variation should also be accounted for by managers interested in the general resilience of the whole species (Chan et al., 2019; López‐Pujol et al., 2012). In fact, with specific attention devoted to connectivity and dispersal rate (Labonne et al., 2008; Razgour et al., 2019), it should be possible to elaborate eco‐evolutionary management plans accounting for small‐scale contrasts in environments. For instance, hybridization could be used as a management tool to perform assisted and controlled gene flow in order to increase evolutionary responsiveness of endangered species facing global changes (Drury & Lirman, 2021; Stelkens et al., 2014).

For many other taxa, contemporary patterns of diversity within and between species are often the result of allopatric evolution (Harrison, 2012), driven by either adaptive or nonadaptive processes (Hendry & Gonzalez, 2008; Mayr, 1970). As a result, reproductive isolation could have potentially evolved between isolated populations using different routes, often involving eco‐evolutionary mechanisms (Schluter, 2000). In that case, genotype‐by‐environment interactions may lead to fitness variations among individuals and dictate the evolutionary outcome of hybridization. This has been observed at intra‐ and interspecific level (Campbell & Waser, 2001; Fraser et al., 2008; Genovart, 2009; Janes & Hamilton, 2017) with studies pointing toward effects of environmental factors, such as temperature (Drury & Lirman, 2021; Krehenwinkel & Tautz, 2013), pH (Fraser et al., 2008), or eutrophication (Vonlanthen et al., 2012) on the fitness of hybrids complex. A growing number of studies are highlighting the need to identify environmental and ecological factors involved in hybridization dynamics (Genovart, 2009; Lindtke et al., 2014; Schwartz et al., 2004), specifically when a combination of environmental factors will influence the maintenance of reproductive barriers between and within species (Janes & Hamilton, 2017). As in our case study, spatial—or even temporal—variations of such factors could be the key to building future management strategies of intraspecific diversity for admixtured gene pools. Indeed, they will be able to address simultaneously several objectives, such as conserving native diversity in some areas (Bohling, 2016; Janes & Hamilton, 2017) and maximizing evolutionary potential as a whole in others, in order to cope with future environmental variation (Nuismer & Gandon, 2008).

## CONFLICT OF INTEREST

We declare no conflict of interest.

## Supporting information

Data S1

Data S2

Data S3

Data S4

Data S5

Data S6

## Data Availability

The data that support the findings of this study are openly available on data.inrae.fr at https://doi.org/10.15454/KB0NL3.
