# Supplementary material for: Genotype‐by‐environment interactions drive the maintenance of genetic variation in a Salmo trutta L. hybrid zone
Source: Evol Appl. 2021 Oct 30;14(11):2698–711. doi: 10.1111/eva.13307 (PMC8591331; doi:10.1111/eva.13307)
Supplement: Supplementary file 1 — Data S1 [file EVA-14-2698-s006.docx]

**Supplementary Material 1: Robe criteria used in lineage characterization and determination of expected genotypic score.**

| **Robe criteria** | **MED** | **HYB** | **ATL** |
| --- | --- | --- | --- |
| **Lateral** **stripes** | Present | Possible | Absent |
| **Spot aureole** | Absent | Possible | Present |
| **Lateral line** | Absent | Partial | Present |
| **Opercular macrostigma** | Present | Possible | Possible |
| **Number of opercular spots** | High | Medium | Low |

Because it was paramount to minimize duration of fish captivity, adults were selected on robe criteria for the fertilization protocol. Indeed, the different genotypic categories (i.e. genotypes: ATL, HYB, MED) can to some extent be phenotypically differentiated based on robe criteria defined by Aparicio et al. (2005). Saint-Pé et al. (2019) provided an in-depth genomic analysis of the genetic bases of these robe criteria, which have already been used as a proxy of lineages characterization for field observations (Gil et al., 2015). For each possible expected genotype, it is noted whether robe criteria are present, possible, partial, or absent. The number of opercular spots is ranged from high to low. Lateral stripes, spot aureole, lateral line, and the number of opercular spots were used in the calculation of our expected genotypic score as described in Gil et al. (2015). Opercular macrostigma was used to validate the attribution to the MED lineage, as it is a compulsory criterion in MED individuals.
