# Supplementary material for: Genotype‐by‐environment interactions drive the maintenance of genetic variation in a Salmo trutta L. hybrid zone
Source: Evol Appl. 2021 Oct 30;14(11):2698–711. doi: 10.1111/eva.13307 (PMC8591331; doi:10.1111/eva.13307)
Supplement: Supplementary file 2 — Data S2 [file EVA-14-2698-s003.docx]

**Supplementary material 2:**

**Relationship between maternal genotypic score and mean egg size.**


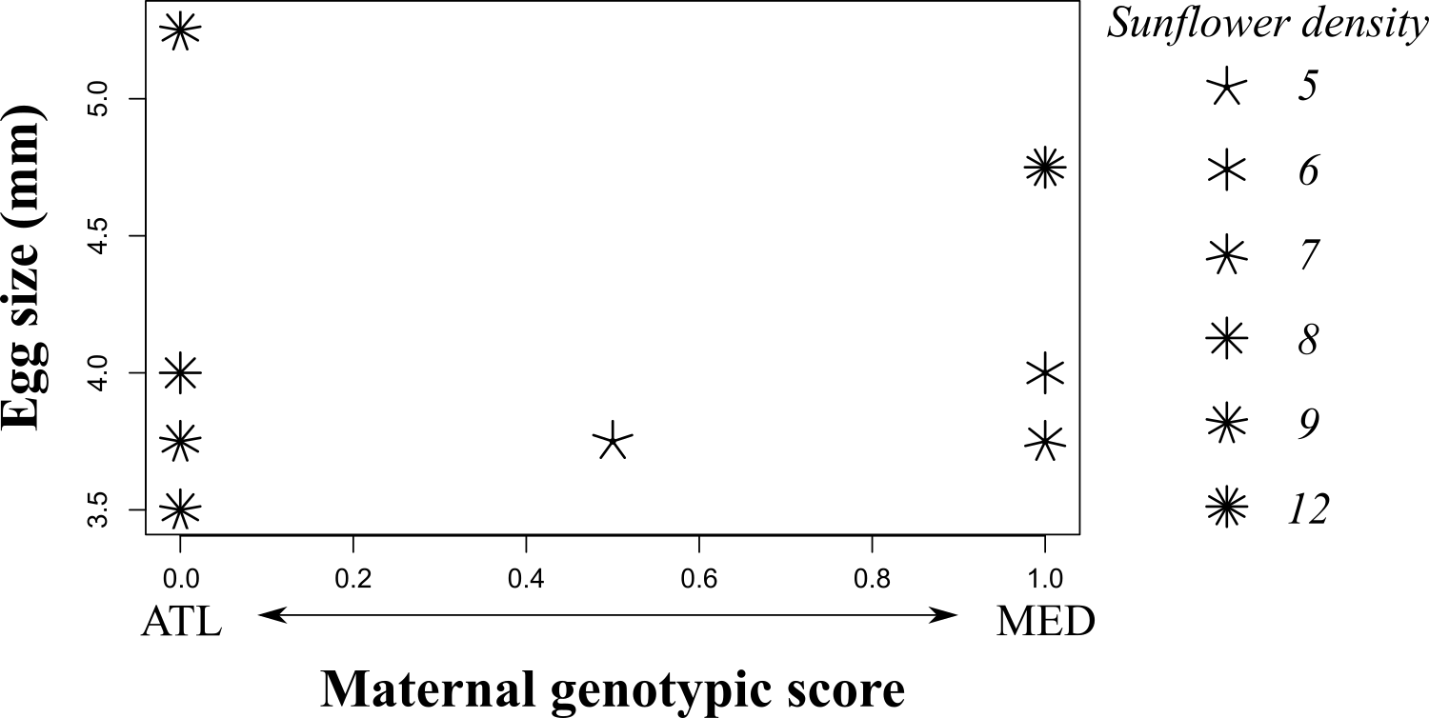


Figure 1: Sunflower plot representing mean egg diameter (mm) within a sub-batch as a function of maternal genotypic score for the 65 recovered incubation boxes. The sunflower density indicates the number of incubation boxes for a given combination of maternal genotypic score and mean egg diameter.
