# Supplementary material for: Genotype‐by‐environment interactions drive the maintenance of genetic variation in a Salmo trutta L. hybrid zone
Source: Evol Appl. 2021 Oct 30;14(11):2698–711. doi: 10.1111/eva.13307 (PMC8591331; doi:10.1111/eva.13307)

**Supplementary Material 3: Experimental design for the fertilization protocol using individuals selected based on their expected genotypes.**

For each of the nine females, three of each expected genotypes (MED, HYB, and ATL), clutches were divided into four batches. Three were destined to be placed in the three thermally contrasted rivers (b, c, and d) and the remaining one (a) was kept to control for egg fertilization in the experimental fish farm (Thonon-les-Bains, INRAE, E74 300-4). Each batch was then divided into three sub-batches (1, 2, 3) in order to be fertilized by semen from either, expected MED, expected HYB, or expected ATL males; making a total of 81 sub-batches for our field experiment.


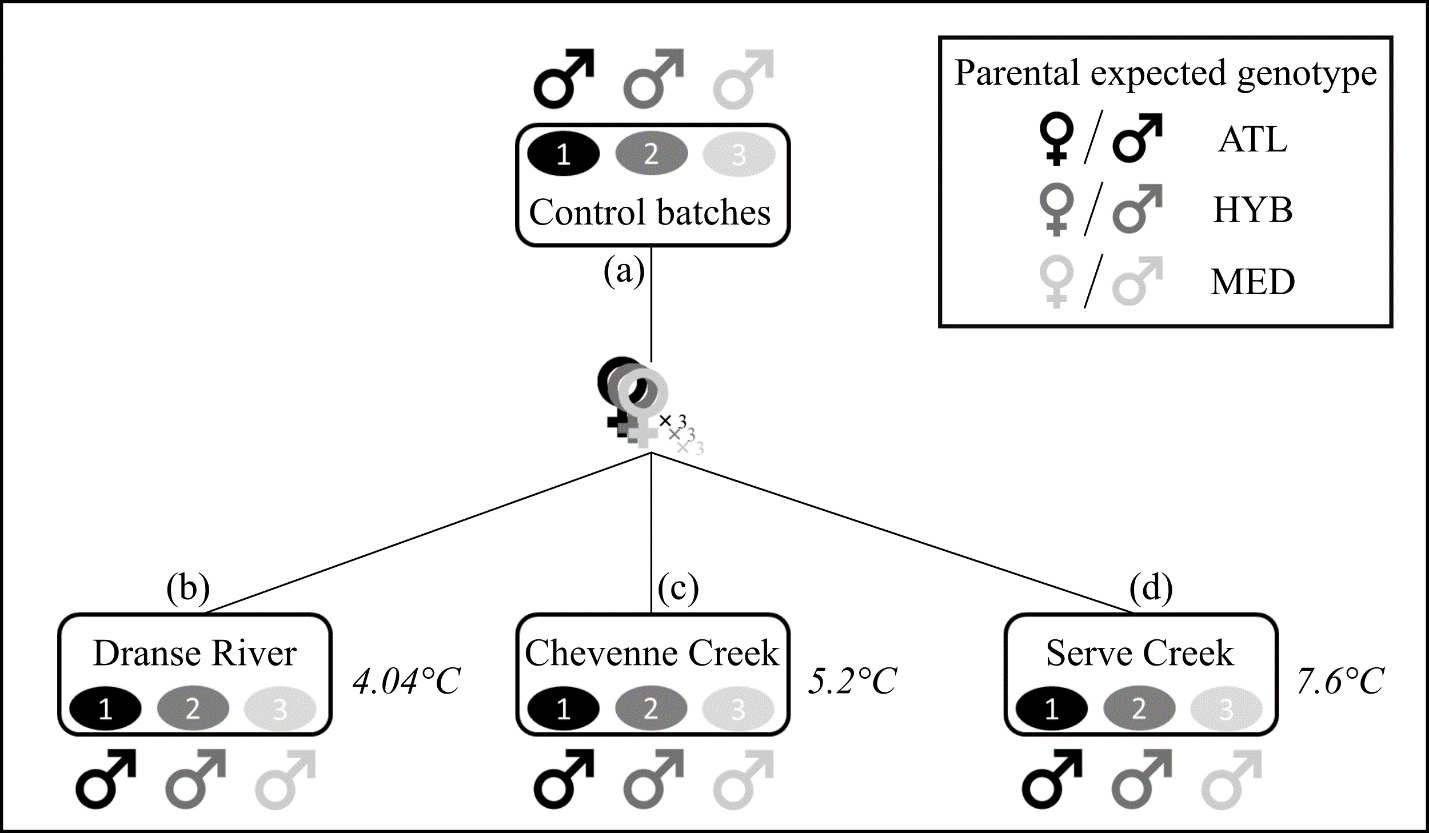

Supplement: Supplementary file 3 — Data S3 [file EVA-14-2698-s001.docx]
