# Supplementary material for: Genotype‐by‐environment interactions drive the maintenance of genetic variation in a Salmo trutta L. hybrid zone
Source: Evol Appl. 2021 Oct 30;14(11):2698–711. doi: 10.1111/eva.13307 (PMC8591331; doi:10.1111/eva.13307)
Supplement: Supplementary file 4 — Data S4 [file EVA-14-2698-s005.docx]

**Supplementary Material 4: Relationship between expected genotype based on robe criteria and ascertained genotypic scores.**

Genotypic scores based on genetic markers were obtained posterior to fertilization and were compared to the phenotypic attribution of an origin using an expected genotypic score determined based on the presence or absence of traits related to robe criteria (see Supplementary Material 1). Both scores were strongly correlated for males and females (r = 0.65, p=0.0004 for males, r = 0.82, p=0.0063 for females, Figure 1) as anticipated (Aparicio et al., 2005; Saint-Pé et al., 2019), which indicated that we did obtain sufficient contrast in genetic origins using robe criteria to organize our crosses, especially regarding pure genotypes since we obtained genetically pure MED individuals.

Looking at the distribution of actual genotypes (Figure 2), some hybrid genotypes were missing, as some of the hybrid expected genotypes (based on robe criteria) turned out to be classified as genetically pure (based on genetic markers). This slightly changed our experimental design, resulting in a lower proportion of crosses involving hybrids individuals (Supplementary Material 5). Nevertheless, it did not impair our results since statistical analyses were based on fish actual genotypic scores only.


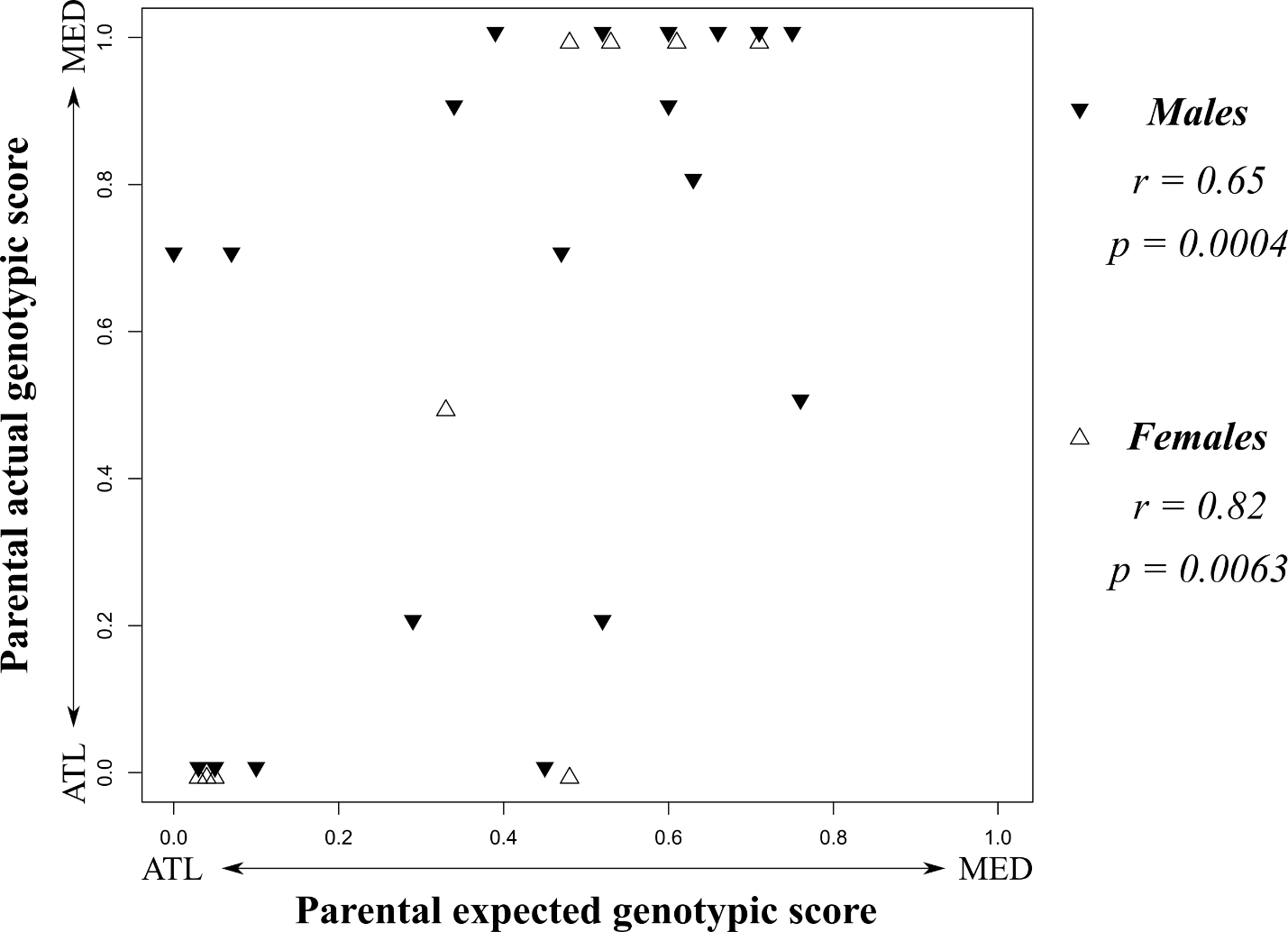


Figure 1: Correlation between parental expected and actual genotypic score. Correlation coefficients (r) and p-values are given separately for males (▼) and females (Δ).


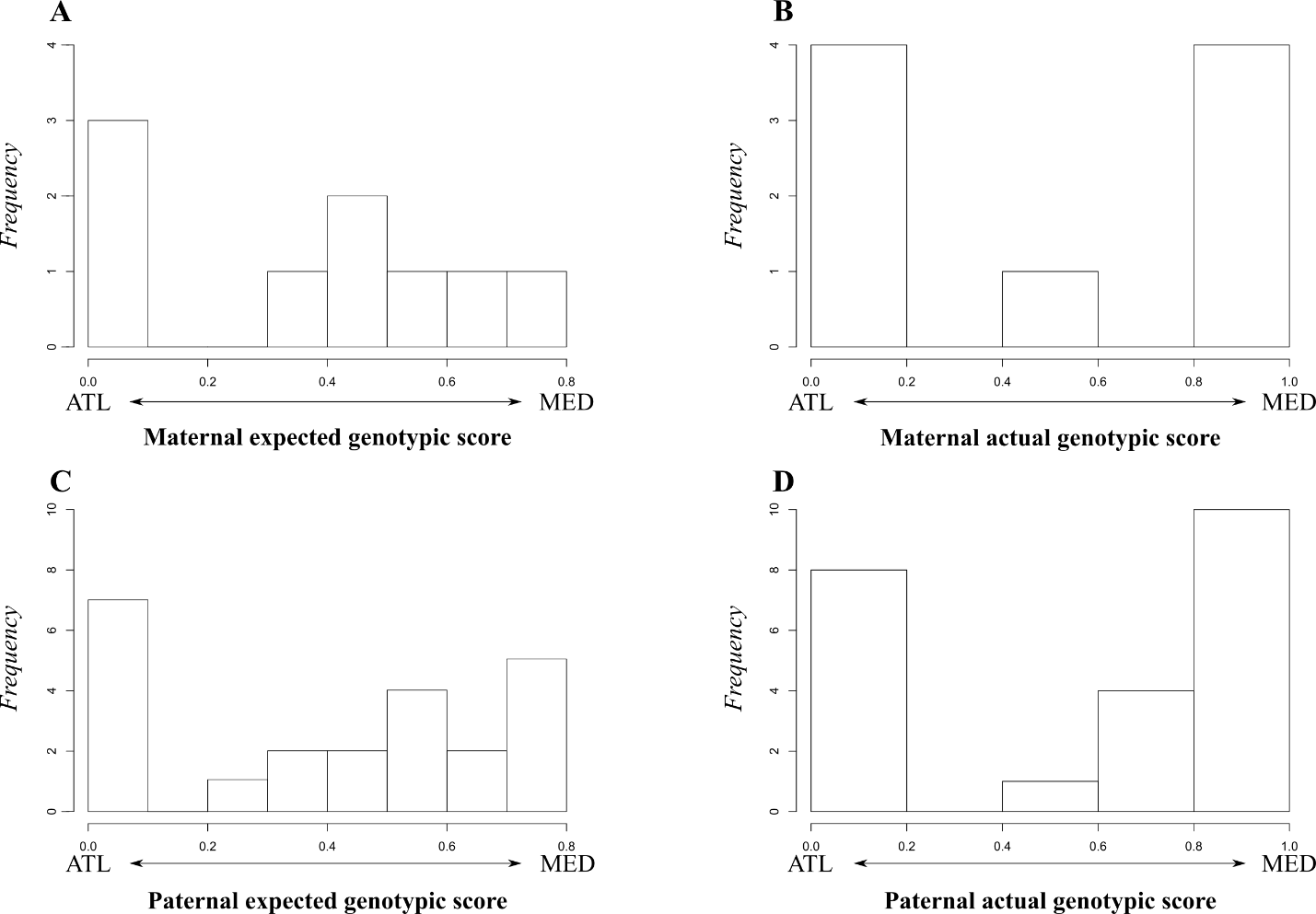


Figure 2: Distribution of (A) maternal expected genotypic score, (B) maternal actual genotypic score, (C) paternal expected genotypic score and (D) paternal actual genotypic score.
