# Supplementary material for: Genotype‐by‐environment interactions drive the maintenance of genetic variation in a Salmo trutta L. hybrid zone
Source: Evol Appl. 2021 Oct 30;14(11):2698–711. doi: 10.1111/eva.13307 (PMC8591331; doi:10.1111/eva.13307)
Supplement: Supplementary file 5 — Data S5 [file EVA-14-2698-s004.docx]

**Supplementary material 5:**

**Remaining parental combination in each river location.**

Due to nest scouring (16 incubation boxes out of 81 were lost), the number of recovered incubation boxes and frequency of each parental genotypes combinations differed between rivers. Some parents could not be represented in some rivers either due to nest scouring or to the lack of gametes for males. Female 8 and males 3, 13, 15, 19, 20, 21, 22 were missing in the Dranse River; males 4, 16, 18, 19 missing in the Chevenne Creek and males 4, 12, 13, 14, 15, 22 missing in the Serve Creek.


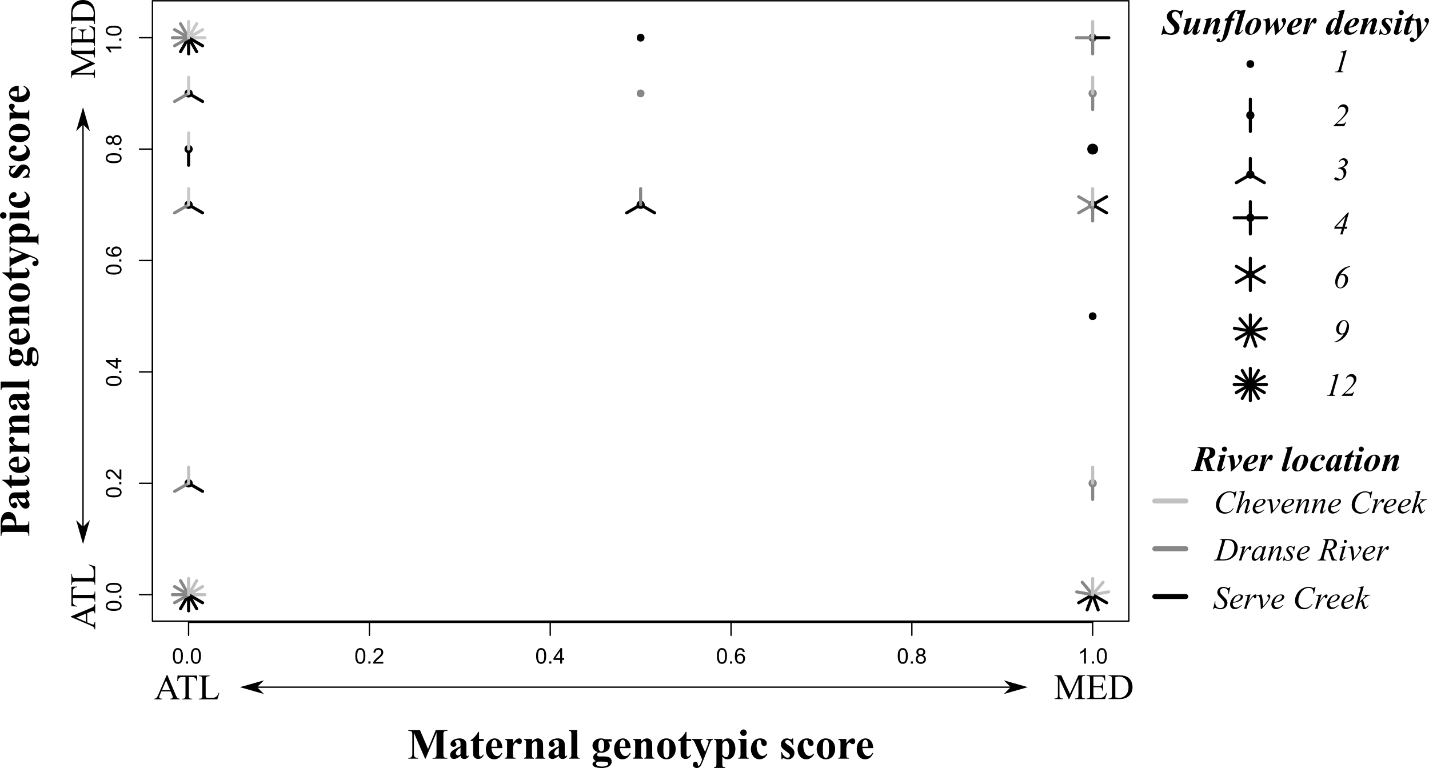


Figure 1: Sun Flower plot representing maternal and paternal genotypic scores combination involved in each remaining crosses for our 65 incubation boxes. The sunflower density indicates how many incubation boxes were recovered per parental combination. Colors of traits represent the river location – Chevenne Creek (light grey), Dranse River (dark grey) and Serve Creek (black) – of each incubation box.
