# Supplementary material for: Genotype‐by‐environment interactions drive the maintenance of genetic variation in a Salmo trutta L. hybrid zone
Source: Evol Appl. 2021 Oct 30;14(11):2698–711. doi: 10.1111/eva.13307 (PMC8591331; doi:10.1111/eva.13307)

**Supplementary material 6:**

**Random effects estimates for each female (A), male (B) and river (C). The box plot represents 2.5, 25, 50, 75 and 97.5% quantiles of the estimates density probability.**


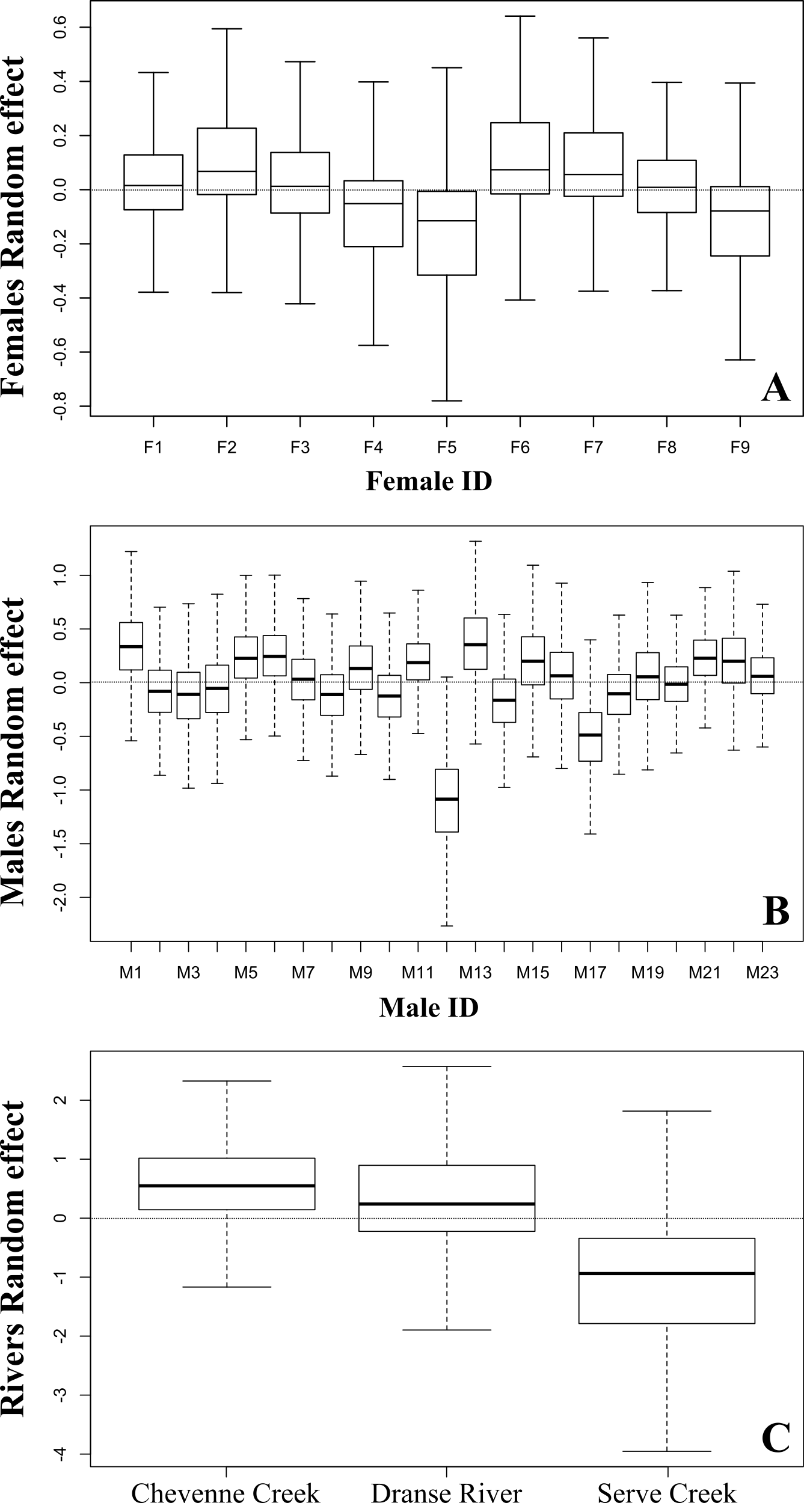

Supplement: Supplementary file 6 — Data S6 [file EVA-14-2698-s002.docx]
